# Cholest-4,6-Dien-3-One Promote Epithelial-To-Mesenchymal Transition (EMT) in Biliary Tree Stem/Progenitor Cell Cultures In Vitro

**DOI:** 10.3390/cells8111443

**Published:** 2019-11-15

**Authors:** Lorenzo Nevi, Daniele Costantini, Samira Safarikia, Sabina Di Matteo, Fabio Melandro, Pasquale Bartolomeo Berloco, Vincenzo Cardinale

**Affiliations:** 1 Department of Translation and Precision Medicine, “Sapienza” University of Rome, 00185 Rome, Italy; daniele.costantini.rm@gmail.com (D.C.); samira.safarikia@uniroma1.it (S.S.); dimatteo.sabina@gmail.com (S.D.M.); 2 Department of General Surgery and Organ Transplantation, Sapienza University of Rome, 0016 Rome, Italy; fabmelan@yahoo.it (F.M.); pasquale.berloco@uniroma1.it (P.B.B.); 3 Department of Medico-Surgical Sciences and Biotechnologies, Polo Pontino, “Sapienza” University of Rome, 04100 Latina, Italy

**Keywords:** senescence, telomerase, biliary tree stem/progenitor cells (BTSCs), primary sclerosing cholangitis (PSC), epithelial-to-mesenchymal transition (EMT), BMP pathway, SHH pathway

## Abstract

Human biliary tree stem/progenitor cells (hBTSCs), reside in peribiliary glands, are mainly stimulated by primary sclerosing cholangitis (PSC) and cholangiocarcinoma. In these pathologies, hBTSCs displayed epithelial-to-mesenchymal transition (EMT), senescence characteristics, and impaired differentiation. Here, we investigated the effects of cholest-4,6-dien-3-one, an oxysterol involved in cholangiopathies, on hBTSCs biology. hBTSCs were isolated from donor organs, cultured in self-renewal control conditions, differentiated in mature cholangiocytes by specifically tailored medium, or exposed for 10 days to concentration of cholest-4,6-dien-3-one (0.14 mM). Viability, proliferation, senescence, *EMT* genes expression, telomerase activity, interleukin 6 (IL6) secretion, differentiation capacity, and *HDAC6* gene expression were analyzed. Although the effect of cholest-4,6-dien-3-one was not detected on hBTSCs viability, we found a significant increase in cell proliferation, senescence, and IL6 secretion. Interestingly, cholest-4.6-dien-3-one impaired differentiation in mature cholangiocytes and, simultaneously, induced the EMT markers, significantly reduced the telomerase activity, and induced *HDAC6* gene expression. Moreover, cholest-4,6-dien-3-one enhanced bone morphogenic protein 4 (Bmp-4) and sonic hedgehog (Shh) pathways in hBTSCs. The same pathways activated by human recombinant proteins induced the expression of EMT markers in hBTSCs. In conclusion, we demonstrated that chronic exposition of cholest-4,6-dien-3-one induced cell proliferation, EMT markers, and senescence in hBTSC, and also impaired the differentiation in mature cholangiocytes.

## 1. Introduction

Primary sclerosing cholangitis (PSC) is a rare idiopathic, heterogeneous, cholestatic liver disease characterized by chronic inflammation of bile ducts, leading to duct fibrosis with associated strictures and liver cirrhosis, bearing an exceptionally high malignancy risk, primarily cholangiocarcinoma (CCA) [1,2,3,4,5]. The aetiopathogenesis of PSC is very complex and not yet well defined [4]. Moreover, PSC remains one of the most complex pathological conditions of clinical hepatology because still, there is no available medical therapy that has been proven effective for the clinical endpoints [3,5]. Previous studies have identified the genetic predisposition, the role of exogenous and endogenous factors responsible for the PSC development in humans [6,7,8].

Moreover, several studies have demonstrated a putative role of oxysterols in the pathogenesis of human PSC [8,9]. Oxysterols promote the inflammation and necrosis, induce immunosuppression, and could enhance colon carcinogenesis [10,11,12]. Oxysterols can bind to the specific nuclear receptor, so they can play a pivotal role in several processes, such as cytotoxicity, mutagenesis, and carcinogenesis [10,13]. Although the biliary epithelium is uninterruptedly exposed to bile flow, there is little knowledge about the oxysterol’s role in the biliary pathophysiology. Haigh and Lee have recently reported that oxysterol cholesta-4,6-dien-3-one was highly presented in human brown pigment gallstones in correlation with abundant bacterial DNA [14]. Their observations suggest that biliary oxysterol could be associated with bacteria presence and could have an important role in the biliary disease development. Recently, it has been highlighted that the occurrence of a bile dysbiosis can have a putative role in patients affected by PSC, but still, an exact relation between dysbiosis and bile composition has not been defined [15].

Previous studies have demonstrated the regeneration capacity of the biliary tree after biliary damage in vitro in mouse models and human tissue analysis [16,17]. Interestingly, we have shown in a previous manuscript that the biliary epithelium regeneration during and after biliary injury was due to activation of biliary tree stem/progenitor cells (BTSCs) localized in peribiliary glands (PBGs) through Wnt or Notch pathways induction [18].

In this paper we have investigated the biological effects of oxysterols in human biliary epithelial cell populations, comprising BTSCs and differentiated cholangiocytes. The results revealed a pleiotropic effect of oxysterols in cell biology, confirming the potential of these molecules to trigger alterations which were observed in human cholangiopathies, especially PSC and CCA.

## 2. Materials and Methods

### 2.1. Human Tissue Sourcing

Human extrahepatic biliary tree tissues (N = 6) were obtained by donor organs from the ‘‘Paride Stefanini’’ Department of General Surgery and Organ Transplantation, Sapienza University of Rome, Rome, Italy. Informed consent was obtained to use tissues for research purposes from our transplant program. All samples derived from adults between the ages of 19 and 73 years. Protocols received the approval of our Institutional Review Board, and processing was compliant with current Good Manufacturing Practice (cGMP). The research protocol was reviewed and approved by the Ethics Committee of Umberto I University Hospital, Rome on 17 June 2010. The reference number of this study is Prot. 541/1. No donor organs were obtained from executed prisoners or other institutionalized individuals. More details about the donor organs are provided in Supplementary Table S1.

### 2.2. Cells Isolation

Human biliary tree stem/progenitor cells were isolated as described in our previous studies [19,20,21,22]. Briefly, extrahepatic biliary tree tissues were washed with DPBS (Gibco #14190-094) and were digested in RPMI 1640 (Gibco # 31870)-based digestion buffer enriched with 0.1% human serum albumin, 1 nM selenium, antibiotics, 300 unit/mL type I collagenase, and 0.3 mg/mL deoxyribonuclease. Digestion was performed at 37 °C for 30 ± 5 min. Then, cells were progressively filtered by sterile 800, 100 and 30 µm strainers to remove greater residues. Cell solution obtained was immunosorted for EpCAM marker by EpCAM antibody-coated microbeads (Miltenyi Biotec Inc., Bergisch Gladbach, Germany #130-061-101) and loaded onto a MACS LS column (Miltenyi Biotec Inc., Bergisch Gladbach, Germany #130-042-401) in the presence of a magnetic field generated by MACS separator. EpCAM+ cells obtained were counted and suspended at a concentration of 500,000 cells/mL in basal medium (Kubota’s medium), and then used as the final cell suspension.

### 2.3. Kubota’s Medium

Kubota’s medium (KM) is a serum-free medium which was studied and developed for survival and growth of endodermal stem/progenitor cells [23]. Details of KM preparation protocol were first reported by Kubota and Reid [23]. According to these studies, KM proved to be successful for hBTSCs [19,20,21,24] and was used as basal condition control in all performed experiments.

### 2.4. Cell Cultures

Approximately 5.2 × 10^4^ cell/cm^2^ (EpCAM^+^ hBTSCs) were seeded in multiwell 6 (JET Biofil, Caton, China #TCP011006) with KM supplemented with fetal bovine serum (FBS) 10% for the first day after cell isolation. After 24 h, the medium was replaced with KM without FBS and it was changed every three days. When the cells obtained the optimal conditions considering morphology and colony formation, they were treated with KM (control condition) or KM added with cholest-4,6-dien-3-one (Gentaur, Milan, Italy #TRC-C431420-2.5) at 0.14 mM [1]. Cells were cultured for 10 days in these conditions in order to mimic the chronic exposure which occurs during human PSC. After 10 days, the cell media were collected and stored at −20 °C, cells were detached by using the trypsin (Gibco # 12604) and were stored at −20 °C in the trizol (Invitrogen, Carlsbad, California, USA #15596026) or ripa (Sigma, St. Louis, Missouri, USA #P5726) for gene or protein analysis, respectively. Furthermore, hBTSCs were cultured in hormonally defined medium specifically tailored to induce the differentiation of hBTSCs toward mature cholangiocytes (CM) [19], or CM supplemented with cholest-4,6-dien-3-one at 0.14 mM for 15 days. The medium was changed every three days and cholest-4,6-dien-3-one was added to CM at day 5 in order to perform a chronical exposure of 10 days.

Finally, in vitro signaling pathway effects on hBTSCs were evaluated by supplementing the KM with the activators and inhibitors, as follows:

- **Hedgehog pathway** [25]: Purmorphamine (1.5 µM, Sigma, St. Louis, Missouri, USA #SML0868), DAPT (1.0 µM, Sigma, St. Louis, Missouri, USA #D5942) and dorsomorphin (2.0 µM, Sigma, St. Louis, Missouri, USA #P5499).
- **BMP pathway** [25,26]: human Bmp-4 (20 ng/mL Sigma, St. Louis, Missouri, USA #B2680), DAPT (1.0 µM, Sigma, St. Louis, Missouri, USA #D5942) and cyclopamine hydrate (0.25 µM, Sigma, St. Louis, Missouri, USA #C4116).

KM was used as basal condition. Pathways induction was performed for 15 days, and the medium was changed every three days. At the end of treatment, cells were detached using trypsin (Gibco, Waltham, Massachusetts, USA # 12604) and stored at −20 °C in trizol (Invitrogen, Carlsbad, California, USA #15596026).

### 2.5. Proliferation and Viability Analysis

hBTSCs were detached using trypsin and counted by trypan blue exclusion assay at several time points (1, 3, and 10 days) to count the cell number in cultures. At day 10, cell viability and proliferation index (population doubling (PD)) were measured by data obtained from trypan blue exclusion assay and the formula described in other studies [19,20]. Values are expressed as mean ± standard deviation. Briefly, equations used for cell viability and PD are reported below respectively:

(1) $$Cell\;viability\;=\;\frac{Cells\;viable}{\left(cells\;viable+cells\;dead\right)}$$

(2) $$PD\;=\;\frac{\log_{10}\left(N\right)-log_{10}\left(Ns\right)}{log_{10}\left(2\right)}$$

where N is the harvested cell number, and Ns is the number of the seeded cells initially plated cell number.

### 2.6. Cell Senescence

Senescence after cholest-4,6-dien-3-one treatment was analyzed by X-Gal test (Sigma, St. Louis, Missouri, USA #CS0030), as described previously [19]. Briefly, cells were incubated with chromogenic X-Gal substrate for 4 h in incubator at 37 °C in the absence of CO_2_. Blue cells were counted by optical microscopy used 10 random fields. Values are expressed as mean ± standard deviation.

### 2.7. RNA Extraction and Quantitative Reverse-Transcription Polymerase Chain Reaction (RT-qPCR) Analysis

Cell RNA extraction was performed as described by Roibaudo et al. [27]. RT-qPCR was performed as described in previous papers [19], a detailed protocol is described in Supplementary Materials and Methods. The sequences used for the analysis are indicated in Supplementary Table S1.

### 2.8. Protein Extraction and Western Blot

Total protein extraction of cultured hBTSC, in basal condition (KM) and pathologic condition (KM + cholest-4,6-dien-3-one was performed using RIPA Buffer, phosphatase inhibitor cocktail (Sigma, St. Louis, Missouri, USA #P5726) at concentration 1:100 and protease inhibitor cocktail (Sigma, St. Louis, Missouri, USA #P8340) at concentration 1:100). Quantification was carried on through the protein assay dye reagent (Bio-Rad, Hercules, California, USA #500-0006) by spectrophotometry analysis at 595 nm, as described in the previous paper [1]. The total protein extract was subjected to 4%–20% SDS-PAGE (Mini-PROTEAN^®^ TGX™ Bio-Rad, Hercules, California, USA #4561094). The resolved proteins were transferred to a 0.2 µm pore-size nitrocellulose membrane (Bio-Rad, Hercules, California, USA #1620146). The membrane was blocked using no-fat milk at 5% in TBST buffer at +4 °C overnight. The following day, block buffer was washed three times using TBST and also anti-hTERT (GeneTex, Irvine, California, USA #GTX124242) was incubated at concentration 1:500 for +4 °C overnight. Finally, the membrane was washed by TBST to eliminate the primary antibody and the membrane was incubate with the secondary antibody, anti-rabbit HRP (Cell Signaling, Danvers, Massachusetts, USA #7074) at concentration 1:2000 for 1 h at room temperature. At the end of incubation, the secondary antibody was washed by TBST and the membrane was incubated with Clarity Western ECL Substrate (Bio-Rad, Hercules, California, USA #1705061) for 3 min before the development of membrane in the darkroom. The densitometric quantization of the Western blot bands was conducted through the ImageJ software. Gene bands were normalized to GAPDH (SAB, College Park, Maryland, USA #41549), as housekeeping control, and expressed as relative quantity of protein. Values are expressed as mean ± standard deviation.

### 2.9. ELISA Assay

Cell medium supernatant at day 10 of culture from two conditions (KM and KM + cholest-4,6-dien-3-one) were harvested and stored at −20 °C. Interleukin 6 (IL6) levels present in the media were measured by ELISA (Cambridge, UK #ab46042) according to the protocol provided by the company. Values are expressed as ng/mL. Values are expressed as mean ± standard deviation.

To confirm gene expression levels obtained by RT-qPCR, the ELISA assays were performed on total protein extraction. Twist 1 (Cusabio, Houston, Texas, USA #CSB-EL025358HU), Twist2 (Mybiosurce, San Diego, California, USA #MBS773864), Snail1 (LSBio, Seattle, Washington, USA #LS-F2317), Snail2 (Biomatik, Cambridge, Ontario, Canada #EKC35582), Zeb1 (Biomatik, Cambridge, Ontario, Canada #EKC36072), and Zeb2 (LSBio, Seattle, Washington, USA #LS-F13506) were measured by human-specific ELISA kit according to protocols provided by companies. Values are expressed as ng/mL. Values are expressed as mean ± standard deviation.

### 2.10. Telomerase Activity Quantification

Telomerase activity was measured by telomerase activity quantification qPCR assay kit (ScienCell, Carlsbad, CA, USA #8928). Approximately 4 × 10^6^ cells for each replicate (N = 6) for both cell culture conditions were extracted and analyzed according to the product protocol. Briefly, cells were detached by trypsin, counted by trypan blue exclusion assay, and pelleted. Cell proteins were extracted by cell lysis buffer supplemented with PMSF 0.1 M in isopropanol and β-mercaptoethanol. After cell protein extraction telomerase reaction was performed as described in the product protocol. Finally, qPCR was done to analyze the telomer production by telomerase. ΔCq was calculated by the following equation:

(3) $$\Delta Cq_{\;}\;=\;Cq_{\left(\;CTRL\;\right)}-\;Cq_{\left(Cholest-4,6-dien-3-one\right)}$$

ΔCq is quantification cycle value obtained from qPCR.

The relative telomerase activity was calculated using the formula described above:

(4) $$Relative\;telomerase\;activity\;=\;2^{-\Delta \mathrm{Cq}}$$

### 2.11. Protein Pathway Analysis

Protein cell extracts were analyzed by RayBiotech Inc. (Peachtree Corners, GA, USA) for Sonic Hedgehog (Shh), bone morphogenic protein 4 (Bmp-4), angiopoietin 2 (Ang-2), Fas pathways for KM and KM supplemented with cholest-4,6-dien-3-one conditions. High-density GS array kits (RayBiotech, Inc., Norcross, GA, USA) allow semi-quantitative determination of the concentrations of human proteins in a single experiment, with detection sensitivity similar to that of a traditional ELISA. The arrays include a 16-well removable gasket that allows the processing of 16 samples on a single slide. The sensitivities of the antibodies in the arrays have been in the order of pg/mL. Values are expressed as mean ± standard deviation.

### 2.12. Statistical Analysis

Values are expressed as the mean ± standard deviation obtained from groups of six samples. statistical analyses were performed by SPSS statistical software (Version 18.0 SPSS Inc., Chicago, IL, USA). Two-tailed Student’s *t*-tests were performed to assess the statistical significance of differences between groups. Statistical significance was set at *p* < 0.05.

## 3. Results

### 3.1. Viability, Proliferation and Senescence after Chronic Cholest-4,6-Dien-3-One Exposure in hBTSC Cultures

#### 3.1.1. Cell Number in hBTSC Cultures

**Figure 1:**
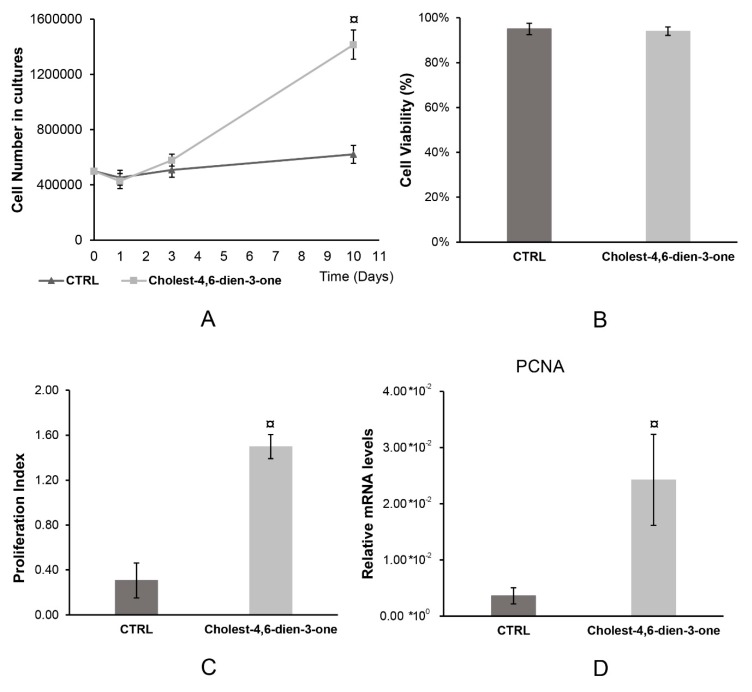
Cholest-4,6-dien-3-one enhance hBTSCs proliferation without affecting cell viability. (**A**) Cell number in cultures determined by trypan blue exclusion assay of hBTSCs cultured in KM added with cholest-4,6-dien-3-one or basal condition (KM). (**B**) Cell viability measured by math equation described above of hBTSCs cultured in KM added with cholest-4,6-dien-3-one or basal condition (KM). (**C**) Proliferation index (PD) calculated by math equation described above of hBTSCs cultured in KM added with cholest-4,6-dien-3-one or basal condition (KM). (**D**) Relative PCNA mRNA level expression analyzed by RT-qPCR of hBTSCs cultured in KM added with cholest-4,6-dien-3-one or basal condition (KM). Data expressed as mean ± SD of N = 6 experiments; ¤ *p* < 0.0001.

hBTSCs were cultured in KM, basal condition, and KM supplemented with oxysterol (cholest-4,6-dien-3-one) for 10 days in order to mimic the PSC chronic injury. At every time point (1, 3, and 10 days) cells were detached and counted by trypan blue exclusion assay. Cells grew in PSC mimic condition for 10 days showed a significant increase of cell number in culture (1′416′000 ± 105′709.03; N = 6; *p* < 0.0001) compared to hBTSCs cultured in basal condition (621′000 ± 65′589.63; N = 6) (Figure 1A). In the early time points (one and three days), no differences were observed between the two culture conditions. This result suggests that in the long period, cholest-4,6-dien-3-one could have a role in cellular proliferation.

#### 3.1.2. Cell Viability

hBTSCs were cultured as described previously. After 10 days of culture, cells were detached and counted both viable and dead cells by trypan blue exclusion assay. At day 10, cells grown in PSC mimic condition (93.98% ± 1.87%) and basal condition (95.04% ± 2.53%) did not show any significant difference in cell viability (N = 6; *p* > 0.05) (Figure 1B). The result achieved could indicate that the cholest-4,6-dien-3-one does not influence cell viability.

#### 3.1.3. Cell Proliferation

Population doubling (PD) was calculated using the equation described in Materials and Methods and the value obtained by trypan blue exclusion assay after 10 days of treatment. At day 10, hBTSC cultured in KM supplemented with cholest-4,6-dien-3-one showed a very significantly enhanced proliferation index (1.50 ± 0.11; N = 6; *p* < 0.0001) when compared to hBTSCs culture in KM (0.31 ± 0.16; N = 6) (Figure 1C). To confirm the enhanced proliferation rate, *PCNA* gene expression was analyzed by RT-qPCR. From our data, hBTSCs cultured for 10 days in KM supplemented with cholest-4,6-dien-3-one showed higher *PCNA* gene level (2.42 × 10^−2^ ± 8.11 × 10^−3^; N = 6; *p* < 0.0001) than cells cultured in KM (3.61 × 10^−3^ ± 1.42 × 10^−3^; N = 6) (Figure 1D). These data observed propose that cholest-4,6-dien-3-one could play a pivotal role in hBTSCs proliferation without affecting cell viability.

#### 3.1.4. Cell Senescence

**Figure 2:**
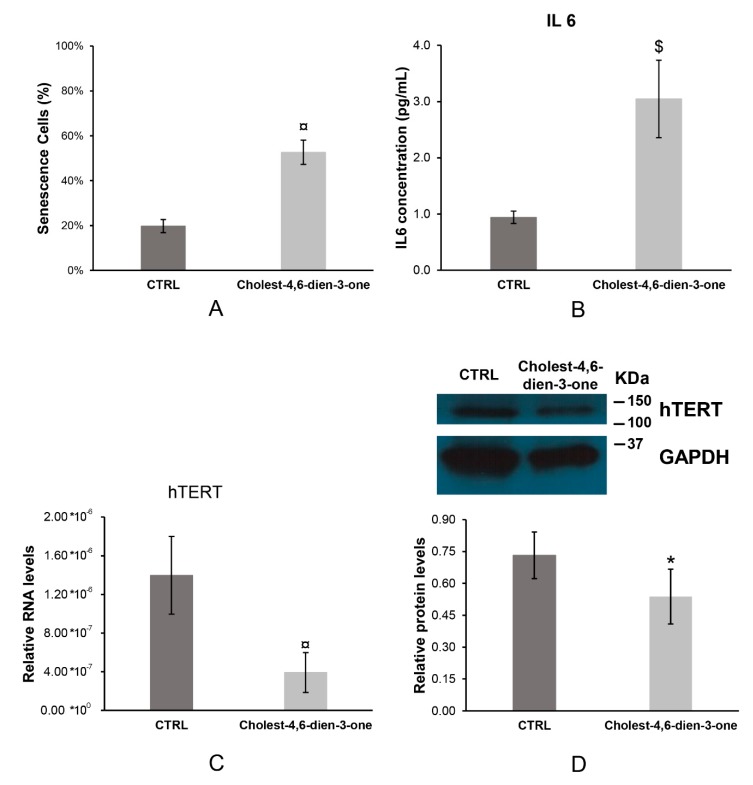
Cholest-4,6-dien-3-one induced cell senescence, impoved IL6 secretion and decreased relative mRNA and protein expression of hTERT. (**A**) Cell senescence in cultures determined by XGAL assay of hBTSCs cultured in KM added with cholest-4,6-dien-3-one or basal condition (KM). (**B**) IL-6 concentration in growth medium measured by ELISA of hBTSCs cultured in KM added with cholest-4,6-dien-3-one or basal condition (KM). (**C**) Relative hTERT RNA levels of expression analyzed by RT-qPCR of hBTSCs cultured in KM added with cholest-4,6-dien-3-one or basal condition (KM). (**D**) Relative hTert protein levels expression analyzed by Western blot of hBTSCs cultured in KM added with cholest-4,6-dien-3-one or basal condition (KM). Data expressed as mean ± SD of N = 6 experiments; * *p* < 0.05; $ *p* < 0.001; ¤ *p* < 0.0001.

Approximal 5.2 × 10^4^ cell/cm^2^ EpCAM^+^ hBTSCs were cultured in KM, basal condition, and KM supplemented with cholest-4,6-dien-3-one for 10 days to mimic the PSC chronic injury. After this period, blue cells were counted and normalized to all cells into the field observed. Cholest-4,6-dien-3-one added to a cell growth medium induced a significant enhancer of senescent hBTSCs (52.64% ± 5.44%; N = 6; *p* < 0.0001) when compared to cell growth in basal condition (19.72% ± 2.90%; N = 6) (Figure 2A). This observation suggests that cholest-4,6-dien-3-one induces high cell senescence after 10 days of chronic cell exposure.

#### 3.1.5. Interleukin 6

Cell growth medium was harvest after 10 days of cell culture in different conditions and stored at −20 °C. Interleukin 6 (IL6) levels were measured by ELISA assay. hBTSCs cultured under chronic exposure of cholest-4,6-dien-3-one showed significantly higher IL6 levels (3.05 ± 0.69 pg/mL; *p* < 0.001; N = 6) when compared to hBTSCs culture in basal condition (0.94 ± 0.11 pg/mL; N = 6) (Figure 2B). As observed in previous studies [28,29,30], our data suggest that high IL6 levels induce cell senescence in hBTSC cultures when cultured in KM supplemented with cholest-4,6-dien-3-one.

#### 3.1.6. Human Telomerase (hTERT)

Telomerase is a riboprotein and so RNA and protein subcomplex were analyzed by RT-qPCR and Western blot, respectively. From our analysis, RNA hTERT relative levels decreased massively in cells exposed to cholest-4,6-dien-3-one for 10 days (3.93 × 10^−7^ ± 2.06 × 10^−7^; *p* < 0.0001; N = 6) compared to hBTSCs cultured in KM for 10 days (1.40 × 10^−6^ ± 4.03 × 10^−7^; N = 6) (Figure 2C). A similar result was observed in hTert protein levels analyzed. hBTSCs cultured in KM supplemented with cholest-4,6-dien-3-one showed a decrease in protein levels (0.54 ± 0.13; *p* < 0.05; N = 6) compared to hTert protein levels detected in cells cultured in KM (0.73 ± 0.11; N = 6) (Figure 2D). This reduction in hTert complex induced telomerase activity decrease of four-fold in cells cultured in KM supplemented with cholet-4,6-dien-3-one compared to cells cultured in basal condition (KM) (Table 1). These data suggest that chronic exposure to Cholest-4,6-dien-3-one in cell cultures induces senescence by reduction of telomerase quantity and activity.

### 3.2. Expression of Stem Cell, Pluripotent Cell and Epithelial-To-Mesenchymal Transition Markers in hBTSCs Chronically Exposed to Cholest-4,6-Dien-3-One

#### 3.2.1. Stem, Pluripotent and Mature cell Markers

**Figure 3:**
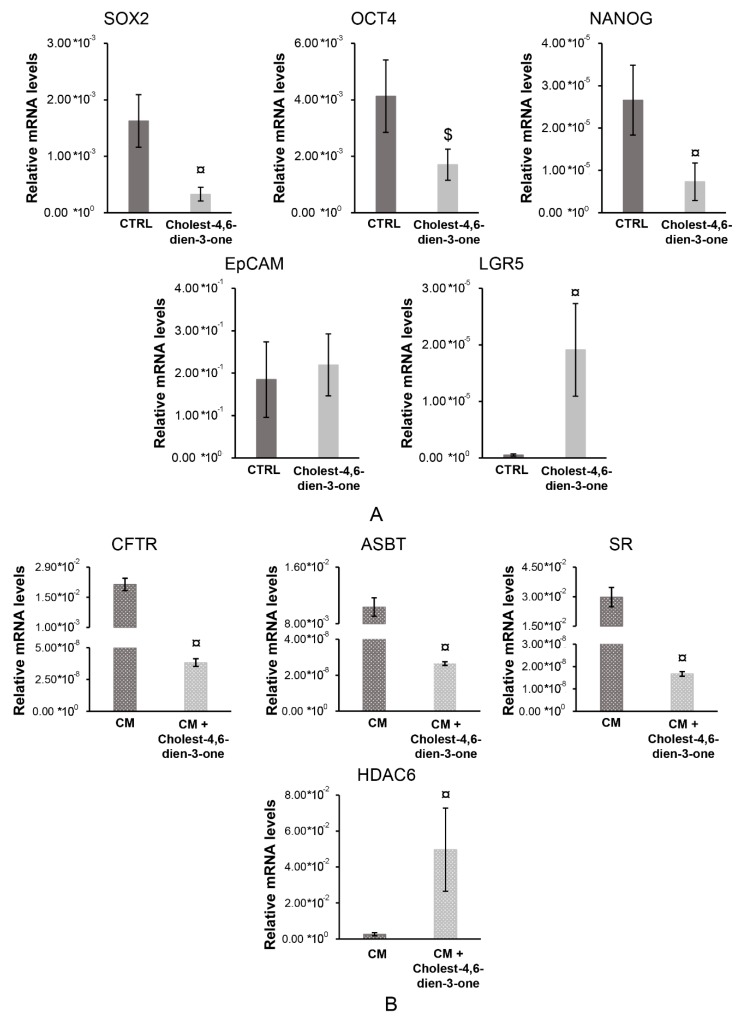
Cholest-4,6-dien-3-one reduced the expression of stem cell markers and blocked the differentiation in mature cholangiocyte. (**A**) Relative gene expression of *SOX2*, *OCT4*, *NANOG*, *EpCAM*, and *LGR5* analyzed by RT-qPCR in hBTSCs cultured in KM added with cholest-4,6-dien-3-one or basal condition (KM). **(B**) Relative gene expression of *CFTR*, *ASBT*, *SR*, and *HDAC6* analyzed by RT-qPCR in hBTSCs cultured in CM added with cholest-4,6-dien-3-one or differentiation medium tailored to mature cholangiocyte (CM). Data expressed as mean ± SD of N = 6 experiments; $ *p* < 0.001; ¤ *p* < 0.0001.

hBTSCs cultured in KM supplemented with cholest-4,6-dien-3-one for 10 days showed a significant reduction in relative mRNA levels of stem cell genes, such as *SOX2* (*p* < 0.0001; N = 6), *OCT4* (*p* < 0.001; N = 6), and NANOG (*p* < 0.0001; N = 6) when compared with hBTSCs cultured in basal condition (Figure 3). At the same time, cells exposed to cholest-4,6-dien-3-one for 10 days showed an increase of LGR5 mRNA levels (*p* < 0.0001; N = 6) when compared to hBTSCs cultured in KM, while only a trend towards an increase was observed in *EpCAM* mRNA levels expression (*p* > 0.05; N = 6) (Figure 3A). Moreover, hBTSCs were expanded in KM preconditioned for five days in CM and thereafter, exposed to CM or CM supplemented with cholest-4,6-dien-3-one for 10 days to mimic cholangiocyte differentiation and an injury along the differentiation, as may happen in bile duct regeneration [18]. From the results obtained, cholest-4,6-dien-3-one supplemented CM markedly impaired cell cholangiocyte differentiation as observed by a significant reduction of mature cell genes *CFTR* (*p* < 0.0001; N = 6), *ASBT* (*p* < 0.0001; N = 6), and *SR* (*p* < 0.0001; N = 6) compared to hBTSCs cultured in CM for 15 days (Figure 3B). As confirmed of cholangiocyte breaking up, e.g., primary cilium remodeling, higher mRNA levels of HDAC6 (*p* < 0.0001; N = 6) were observed in cells cultured in CM added with cholest-4,6-dien-3-one than cells cultured in CM (control) (Figure 3B). Taken together these results suggest that cholest-4,6-die-3-one could induce stem cells to become putative trans-amplifying compartment cells and, at the same time, could block the differentiation in mature cholangiocytes.

#### 3.2.2. Markers of Epithelial-Mesenchymal-Transition

**Figure 4:**
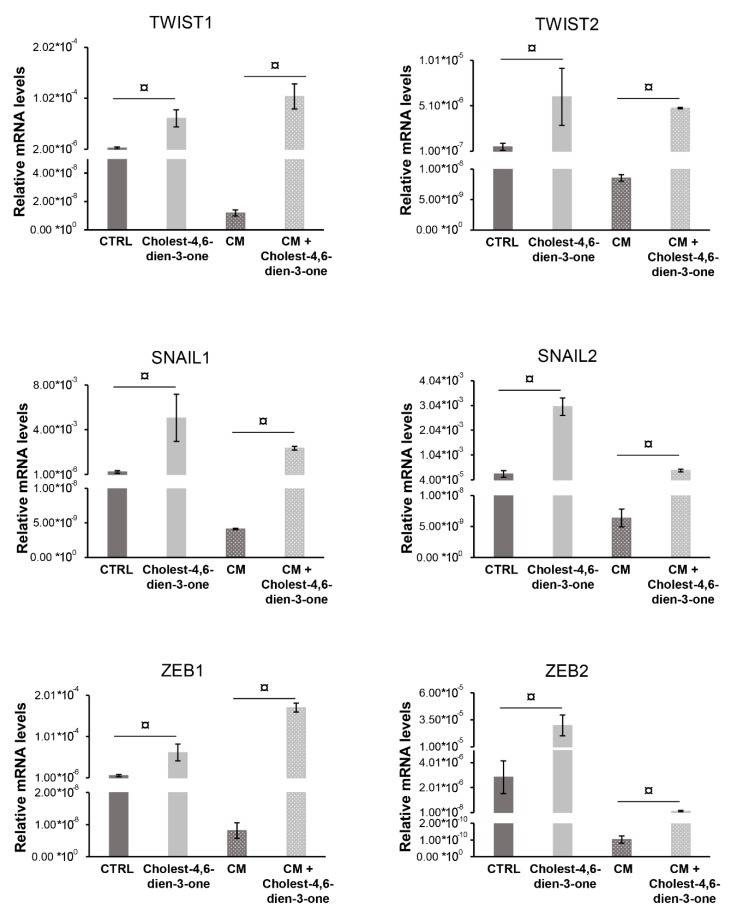
Cholest-4,6-dien-3-one enhanced the EMT markers analyzed by RT-qPCR. Relative gene expression of EMT markers (*TWIST1*, *TWIST2*, *SNAIL1*, *SNAIL2*, *ZEB1*, and *ZEB2*) examined by RT-qPCR of hBTSCs cultured in basal condition (KM), KM supplemented with cholest-4,6-dien-3-one, differentiation tailored cholangiocyte medium (CM) or CM added with cholest-4,6-dien-3-one. Data expressed as mean ± SD of N = 6 experiments; ¤ *p* < 0.0001.

**Figure 5:**
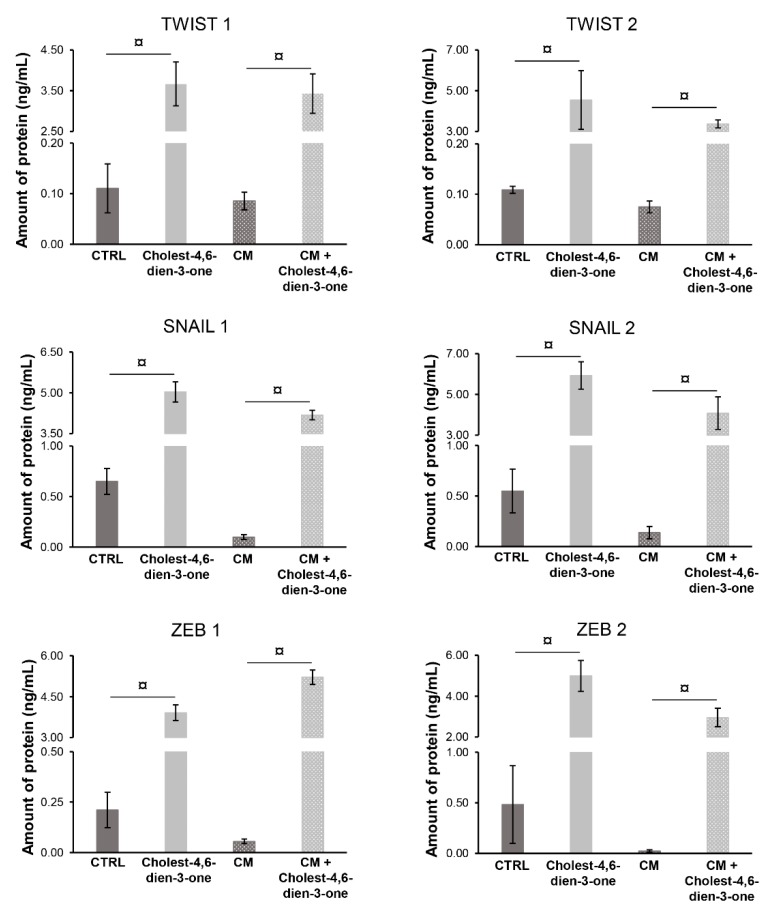
Cholest-4,6-dien-3-one enhanced the EMT markers analyzed by WB. Protein levels of EMT markers (TWIST1, TWIST2, SNAIL1, SNAIL2, ZEB1, and ZEB2) examined by WB of hBTSCs cultured in basal condition (KM), KM supplemented with cholest-4,6-dien-3-one, differentiation tailored cholangiocyte medium (CM) or CM added with cholest-4,6-dien-3-one. Data expressed as mean ± SD of N = 6 experiments; ¤ *p* < 0.0001.

Cholest-4,6-dien-3-one supplemented to KM for 10 days markedly enhanced the relative mRNA levels of *EMT* genes such as *TWIST1* (*p* < 0.0001; N = 6), *TWIST2* (*p* < 0.0001; N = 6), *SNAIL1* (*p* < 0.0001; N = 6), *SNAIL2* (*p* < 0.0001; N = 6), *ZEB1* (*p* < 0.0001; N = 6), and *ZEB2* (*p* < 0.0001; N = 6) when compared with the relative mRNA levels of hBTSCs cultured in basal condition (Figure 4). Furthermore, hBTSCs undergoing cholangiocyte differentiation in vitro when chronically exposed to cholest-4.6-dien-3-one showed a significative increase of EMT mRNA levels genes such as *TWIST1* (*p* < 0.0001; N = 6), *TWIST2* (*p* < 0.0001; N = 6), *ZEB1* (*p* < 0.0001; N = 6), *ZEB2* (*p* < 0.0001; N = 6), *SNAIL1* (*p* < 0.0001; N = 6), and *SNAIL2* (*p* < 0.0001; N = 6) when compared with cells cultured in CM alone (Figure 4). To confirm the qPCR data obtained, protein levels of the same markers were analyzed by ELISA. hBTSCs cultured in KM added with cholest-4,6-dien-3-one showed a significant increase of protein levels TWIST1 (*p* < 0.0001; N = 6), TWIST2 (*p* < 0.0001; N = 6), SNAIL1 (*p* < 0.0001; N = 6), SNAIL2 (*p* < 0.0001; N = 6), ZEB1 (*p* < 0.0001; N = 6), and ZEB2 (*p* < 0.0001; N = 6) when compared to cells cultured in basal condition (KM) (Figure 5). Similar results were observed in cells differentiated with CM or cultured with CM supplemented with cholest-4,6-dien-3-one. In fact, hBTSCs cultured in CM under chronical explosion of cholest-4,6-dien-3-one showed markedly enhanced TWIST1 (*p* < 0.0001; N = 6), TWIST2 (*p* < 0.0001; N = 6), SNAIL1 (*p* < 0.0001; N = 6), SNAIL2 (*p* < 0.0001; N = 6), ZEB1 (*p* < 0.0001; N = 6), and ZEB2 (*p* < 0.0001; N = 6) protein levels compared to protein levels in cells cultured in CM. Results obtained suggest that cholest-4,6-dien-3-one promotes the EMT in hBTSCs chronically exposed, and interestingly, results on differentiation suggest that cholest-4,6-dien-3-one alters the physiological cell differentiation of hBTSCs in cholangiocytes and induces gene expression of *EMT* genes.

### 3.3. Cholest-4,6-Dien-3-One Enhanced the Bmp and Shh Pathway Expression in hBTSCs Treated for 10 Days

#### 3.3.1. Cell Pathways Induction to Cholest-4,6-Dien-3-One

**Figure 6:**
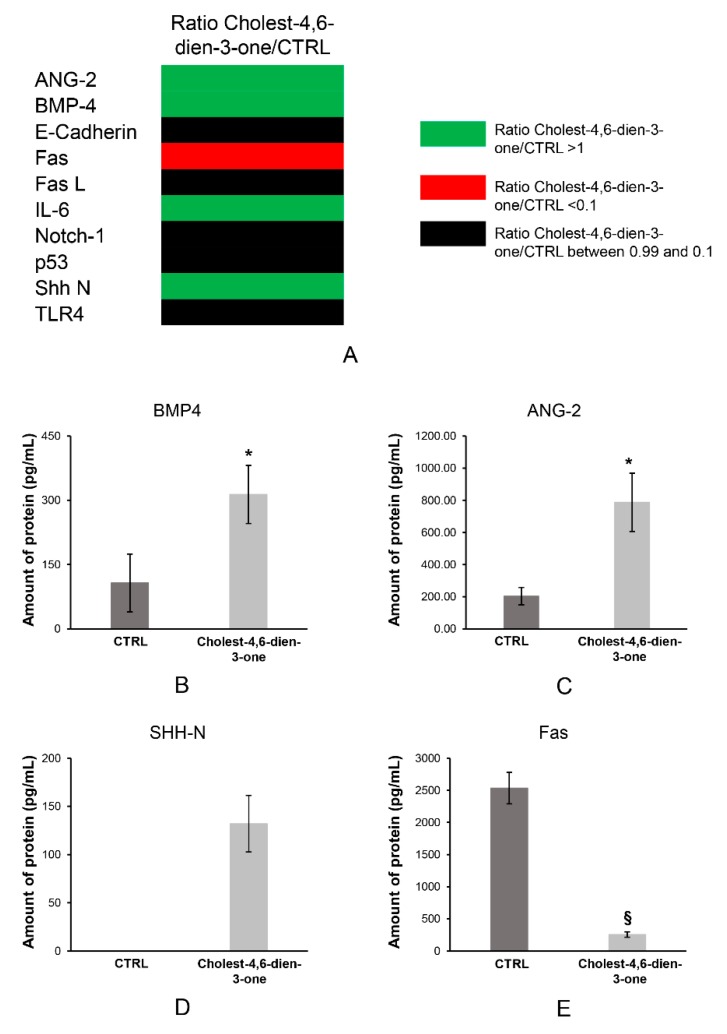
Cholest-4,6-dien-3-one enhance Bmp-4 and Ang-2 pathways, at the same time induced Shh pathway and reduced the expression of Fas. (**A**) Ratio of protein parthways analysed by RayBiotech Inc. in hBTSCs cultured in KM added with cholest-4,6-dien-3-one vs basal condition (KM). (**B**) Amount of Bmp-4 protein in hBTSCs cultured in KM added with cholest-4,6-dien-3-one or basal condition (KM). (**C**) Amount of Ang-2 protein in hBTSCs cultured in KM added with cholest-4,6-dien-3-one or KM. (**D**) Amount of Shh protein in hBTSCs cultured in KM added with cholest-4,6-dien-3-one or KM. (**E**) Amount of Fas protein in hBTSCs cultured in KM added with cholest-4,6-dien-3-one or KM. Data expressed as mean ± SD of N = 3 experiments; * *p* < 0.05; § *p* < 0.01.

Protein extractions from hBTSCs cultured in KM or KM added cholest-4,6-dien-3-one underwent to pathway analysis by RayBiotech Inc. As reported in Figure 6A, the company analyzed a specific protein panels, including Ang-2, Bmp-4, E-Cadherin, Fas, FasL, IL-6, Notch-1, p53, Shh N, and Trl4. Results showed a significant increase in protein amount of Bmp-4 (CTRL: 106.89 ± 67.63 pg/mL; cholest-4,6-dien-3-one: 313.68 ± 67.74 pg/mL; N = 3: *p* < 0.05) (Figure 6B) and Ang-2 (CTRL: 203.75 ± 54.35 pg/mL; cholest-4,6-dien-3-one: 787.31 ± 181.56 pg/mL; N = 3: *p* < 0.05) (Figure 6C) in hBTSCs cultured in KM supplemented with cholest-4,6-dien-3-one with respect control cells. At the same time, Shh N-domain was observed only in hBTSCs exposed for 10 days at cholest-4,6-dien-3-one (132.00 ± 29.46 pg/mL; N = 3) and undetectable in cells cultured in basal condition (Figure 6D). Moreover, the presence of cholest-4,6-dien-3-one in KM induced a significant reduction of Fas receptor (253.48 ± 43.03 pg/mL; N = 3; *p* < 0.01) when compared to the same cells cultured in KM (2535.09 ± 245.57 pg/mL; N = 3) (Figure 6E). The data obtained suggest that cholest-4,6-dien-3-one could play a pivotal role in tumor insurgency, inducing EMT pathway activation and a tumor escape strategy by reduction of Fas receptor.

#### 3.3.2. Bmp-4 and Shh Pathways Stimulated in hBTSCs

**Figure 7:**
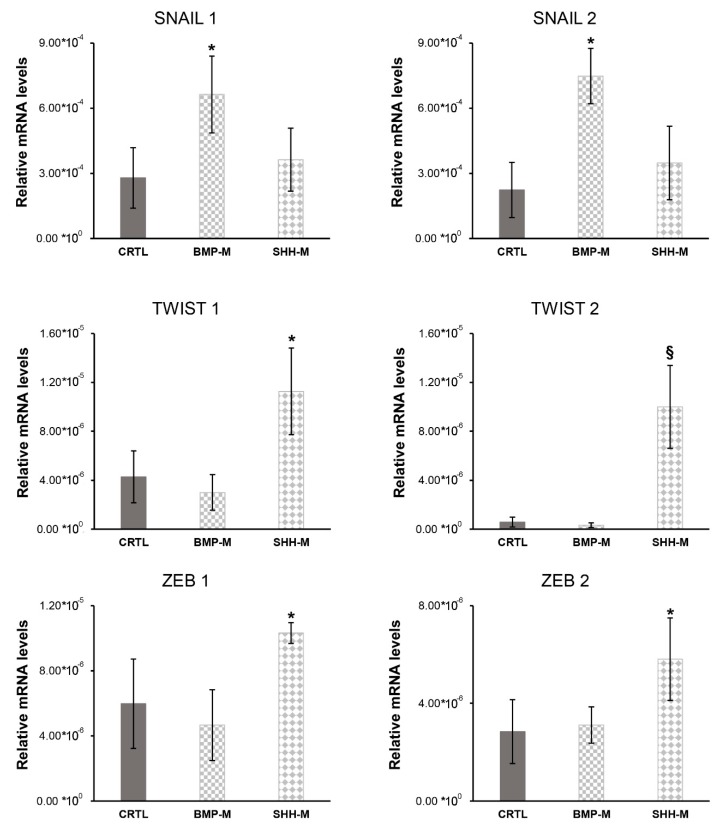
BMP-4 and SHH protein activation pathways induced the expression of *EMT* markers. Relative gene expression of *EMT* markers *TWIST1*, *TWIST2*, *SNAIL1*, *SNAIL2*, *ZEB1*, and *ZEB2*) measured by RT-qPCR of hBTSCs cultured in basal condition (KM), BMP-M or SHH-M. Data expressed as mean ± SD of N = 6 experiments; * *p* < 0.05; § *p* < 0.01.

hBTSCs were cultured in a specific medium inducing Bmp (BMP-M) or Shh (SHH-M) pathway for 15 days. After this period, mRNA was extracted, and gene expression was analyzed by RT-qPCR. From our results, we observed a significative increase in SNAIL1 (N = 6; *p* < 0.05) and SNAIL2 (N = 6; *p* < 0.01) mRNA levels when cells were cultured in BMP-M respect to hBTSCs cultured in KM (Figure 6A). Simultaneously, hBTSCs cultured in SHH-M showed an increase of mRNA levels of TWIST1 (N = 6; *p* < 0.05), TWIST2 (N = 6; *p* < 0.01), ZEB1 (N = 6; *p* < 0.05) and ZEB2 (N = 6; *p* < 0.05) when compared to hBTSCs culture in basal condition (KM) (Figure 7). These results suggest that activation of Bmp and Shh pathways in hBTSCs induces the expression of *EMT* gene markers.

## 4. Discussion

We have investigated the role of a specific oxysterol (cholest-4,6-dien-3-one), implicated with PSC and other cholangiopathies pathogenesis, in the modulation of the biological properties of hBTSCs and differentiated cholangiocytes in vitro. The results obtained in this study have led to several conclusions: (1) Cholest-4,6-dien-3-one increased cell proliferation of hBTSCs without any effect on the cell viability, (2) at the same time cholest-4,6-dien-3-one induced the senescence in hBTSCs treated for 10 days, reducing telomerase expression of protein and mRNA and decreasing telomerase activity. (3) Moreover, cells cultured in KM supplemented with cholest-4,6-dien-3-one secreted high levels of IL6 which enhances the senescence in a cell population. (4) Interestingly, cholest-4,6-dien-3-one induced *EMT* gene expression when added in hBTSC cultures under self-renewal condition (KM) or cholangiocyte differentiation (CM) conditions, affecting, in the second case, the mature cholangiocyte differentiation. (5) Finally, cholest-4,6-dien-3-one promoted the enhancer in Bmp-4 and Shh pathways and at the same time reduce the *Fas* expression. (6) As a confirmation of this, we observed an activating of Bmp-4 or Shh pathways in hBTSC like induction of *EMT* gene markers.

PSC is one of chronic inflammatory cholangiopathies frequently complicated by CCA. Interestingly, significant proliferation of hBTSCs, expansion of PBGs, and dysplasia were observed in PSC [31]. Further results from our group indicated that all CCAs emerging in PSC patients were mucin-producing tumors characterized by PBG involvement and high expression of stem/progenitor cell markers. CCA cells were characterized by a higher expression of epithelial-to-mesenchymal transition (EMT) traits and the absence of primary cilia compared to bile ducts and PBG cells in controls and patients with PSC [1].

Although etiologic factors associated with PSC are largely unknown, biologically relevant endogenous (e.g., oxysterols) and exogenous (e.g., LPS from bacterial translocation) molecules present in the bile have been tested as stressors to mimic cholangiocyte damage in PSC [32,33]. In general, oxysterols were demonstrated to induce cholangiocyte apoptosis, to perpetuate inflammation in cholangiopathies, and to display mutagenic and carcinogenic properties [32,33].

As observed in patients affected by PSC in previous investigations [1], our results demonstrated that in vitro cholest-4,6-dien-3-one induced a proinflammatory behavior observed as high proliferation rate and, at the same time, hBTSCs underwent in senescence due to the cholest-4,6-dien-3-one exposure [32,33]. Moreover, cholest-4,6-dien-3-one was able to induce the expression of EMT markers like *TWIST1*, *TWISTI2*, *SNAIL1*, *SNAIL2*, *ZEB1*, *ZEB2*, and simultaneously enhanced the *histone deacetylase 6* (*HDAC6*) expression, and impaired the cholangiocyte differentiation inhibiting the primary cilium expression [34]. HDAC6 is an important histone protein responsible for the deacetylation of lysine residues on the N-terminal part of the core histones. Deacetylation of histones is an important process for epigenetic repression and plays a pivotal role in several biological functions, such as transcriptional regulation, cell cycle progression, and developmental events. The overexpression of histone deacetylase 6 was recognized as a sign of migration phenotype and tumor invasion in hepatocellular carcinoma and CCA, while its inhibition proved evident effects on the restoration and expression of the cilium in cholangiocytes and in tumor decay [34]. As demonstrated in a previous paper [1], hBTSCs exposed to LPS or to oxysterols showed a partial loss of primary cilia, while, in human mucinous CCA cells we observed mostly the absence of primary cilia. Notably, hBTSCs exposed to LPS and to oxysterols and CCA cells showed higher expression of *LC3* and *p62*, markers of activated autophagy pathway [1]. Moreover as demonstrated in previous papers [35,36,37], hBTSCs possess self-renewal properties and multi-potential differentiation capabilities. Furthermore, a subpopulation of these cells expresses pluripotent stem cell markers [36]. Notably, several stressors, mainly associated with inflammation, induced differentiation of pluripotent stem cells through the perturbation of the unsaturated metabolome which characterizes stem cells [38]. Here, the reduction of pluripotent stem cell markers and the induction of *EMT* genes replicate the effect of stressors observed elsewhere [38].

The *EMT* gene expression and cilium disarrangement are consistent with PBG hyperplasia to tumor development described in patients with PSC [1]. Deciliated hBTSCs could be more susceptible to neoplastic transformation, thus candidate cell of origin for pCCA and dCCA in patients [39]. In severe liver injury, hepatocyte proliferation is an impaired feature of human chronic liver disease [40,41,42,43,44,45]. Telomere shortening and replicative senescence of mature hepatocytes and the arrest of the hepatocyte cell cycle due to specific insults are the main biologic events occurring in the chronic liver disease condition [46,47,48]. Many studies have reported that senescence microenvironment could enhance tumor insurgence [49,50,51]. The telomerase complex comprises TERT (telomerase reverse transcriptase), TERC (telomerase RNA template), dsykerin, NOP10, reptin, pontin, and other factors that are yet to be identified. Telomere lengthening is the canonical function of telomerase. Embryonic stem cells (ESCs) express high levels of telomerase activity that are required to maintain telomere length [52]. Here, we showed that cholest-4,6-dien-3-one reduces telomerase expression and activity in hBTSCs treated for 10 days with oxysterols. Inflammation has been linked to exhaustion of self-renewal in stem cells. Previous works demonstrated that prolonged exposure to oxysterols triggered hBTSC proliferation, increased NF-κB pathway activation and senescence levels, at the same time, a high *pIκB-α/pNF-κB* expression has been observed in PBGs [1,32], suggesting the acquisition of a senescence-associated secretory phenotype (SASP) and the existence of a proinflammatory loop. The stimulation of hBTSCs with oxysterols mimicked the senescence-associated secretory phenotype observed in PSC patients [32]. Here we showed increased interleukin 6 (IL-6) production and secretion in hBTSCs in vitro. In previous studies, a strong link between IL-6 secretion and senescence was demonstrated [28,29,30]. One of the limitation of our study is the lack of a detailed analysis of the SASP profile of cells treated with cholest-4,6-dien-3-one and untreated ones. 

Here, we demonstrated that a putative pro-inflammatory mediator affects telomerase activity which is associated with a senescence-associated secretory phenotype and to proinflammatory loop, leading to the expression of *EMT* markers, and tumor insurgence. Recently, IL-6 and programmed death-1-ligand 1 (PD-L1) antibody blockade combination therapy decreases pancreatic ductal adenocarcinoma (PDAC) tumor progression and increases the percentage of intratumoral effector T cells [53]. Our in vitro data are consistent with the induction of potentially tumor escape strategy in hBTSCs exposed chronically to oxysterols, since cells showed a decrease of Fas in this condition. Riccio et al. demonstrated previously an unexpected modulation of T-cells by hBTSCs through the Fas/Fas-ligand pathway [54,55].

The simultaneous expression of *sonic hedgehog* (*Shh*) and bone morphogenic protein 4 (Bmp-4) pathways was inducted by chronic exposure to cholest-4,6-dien-3-one in hBTSCs. Moreover, the induction of each discrete pathway by specific media supplemented with agonists induced in hBTSCs the expression of *EMT* markers. Interestingly, the induction of Shh or Bmp-4 pathway by agonists was less effective than cholest-4,6-dien-3-one stimulation, when both pathways are activated simultaneously, suggesting that they could play a synergic role in EMT induction in hBTSCs. Many studies have indicated that tumors altered these pathways [56,57,58]. In previous works, both PSC/CCA and PSC samples were characterized by an extremely high expression of *glioma-associated oncogene 1* (*Gli-1*; the effector of the sonic hedgehog pathway) by PBG cells, and neoplastic PBGs without primary cilia maintained high expression of *Gli-1*, suggesting a noncanonical activation of the hedgehog pathway [59,60]. Based on these results, we investigated the activation of these pathways in relation with the induction of *EMT* genes in hBTSCs. In support of our results, many previous studies can be mentioned. In particular, Shh activation modulates EMT of proliferating cholangiocytes of ductular reaction in the course of injuries associated with biliary fibrosis [61]. Similarly, Bmp activation was demonstrated previously to characterize the EMT process in the non-transformed mammary epithelial cell line [62]. However, further analysis should be performed by a specific antagonist to confirm the role of these pathways in the observed effects of oxysterols in hBTSCs.

Moreover, hBTSCs culture in chronic exposure condition showed a decrease of Fas and improving angiopoietin 2 (ANG-2) levels. Fas reductions could suggest that hBTSCs are developing a tumor escape strategy as already analyzed in our previous work in human CCA primary cell lines [54]. This tumor escape strategy has been extensively studied in several tumors [63,64,65,66,67]. Furthermore, the ANG-2 is involved with VEGF to facilitate cell proliferation and migration of endothelial cells [68]. Recently, authors have suggested that ANG-2 could be involved in the regulation of endothelial integrity and inflammation [69].

The major limitation of our study is the lack of mouse models. In fact, to recapitulate the effect of oxysterols on cholangiocytes and hBTSCs, the administration by diet or injection in mouse models may not be appropriate since the concentration in the bile of such molecules is affected by several factors. What has been previously demonstrated by other authors in synthesis is that chronic liver diseases, metabolic alterations associated with obesity, modulate bile composition and are associated with increased levels of biliary oxysterols [70].

Taken together, our results supported the hypothesis that hBTSCs chronically exposed to cholest-4,6-dien-3-one were affected by an inflammatory microenvironment able to provide a permissive milieu for CCA insurgence. Furthermore, the collected data supported the role of hBTSCs in the pathogenesis of PSC and reproduced the changes previously observed in vivo in patients [1].

## 5. Conclusions

We demonstrated cell modification due to the chronical exposition of cholest-4,6-dien-3-one in hBTSC cultures regarding cell proliferation, senescence, *EMT* markers expression and Shh and Bmp-4 pathways activation. Moreover, we demonstrated that cholest-4,6-dien-3-one could impair hBTSC differentiation tailored in mature cholangiocytes. In the human biliary pathology, proliferation, loss of primary cilium, acquisition of EMT features, and senescence are key processes contributing to the transformation and molecule secretion triggering biliary inflammation. Our previous study demonstrated the hBTSCs contribute to PSC insurgency. Based on our data, we suggest that cholest-4,6-dien-3-one could modify the biliary homeostasis by acting on hBTSCs. However, the major limitations of this study is the lack of data obtained from experimental mouse models of cholestasis and from clinical investigations in patients affected by cholangiopaties. 

## Figures and Tables

**Table 1:** Cholest-4,6-dien-3-one induced a four-fold reduction of telomerase activity in hBTSC cultures when compared to hBTSCs cultured in basal condition (KM). ΔCq is quantification cycle value obtained from qPCR.

Sample	Ct	ΔCq	Activity hTERT	Mean & SD
KM 1	17.21	-2.32	4.99	4.40
KM 2	16.86	-2.42	5.35	0.95
KM 3	17.43	-2.21	4.63	
KM 4	17.94	-1.57	2.97	
KM 5	17.06	-2.31	4.96	
KM 6	17.63	-1.81	3.51	
Cholest-4,6-dien-3-one 1	19.53			
Cholest-4,6-dien-3-one 2	19.28			
Cholest-4,6-dien-3-one 3	19.64			
Cholest-4,6-dien-3-one 4	19.51			
Cholest-4,6-dien-3-one 5	19.37			
Cholest-4,6-dien-3-one 6	19.44			

## Supplementary Materials

The following are available online at https://www.mdpi.com/2073-4409/8/11/1443/s1, Table S1: Primers for qPCR.
