# Supplementary figures and images for: Cholest-4,6-Dien-3-One Promote Epithelial-To-Mesenchymal Transition (EMT) in Biliary Tree Stem/Progenitor Cell Cultures In Vitro
Source: Cells. 2019 Nov 15;8(11):1443. doi: 10.3390/cells8111443 (PMC6912632; doi:10.3390/cells8111443)

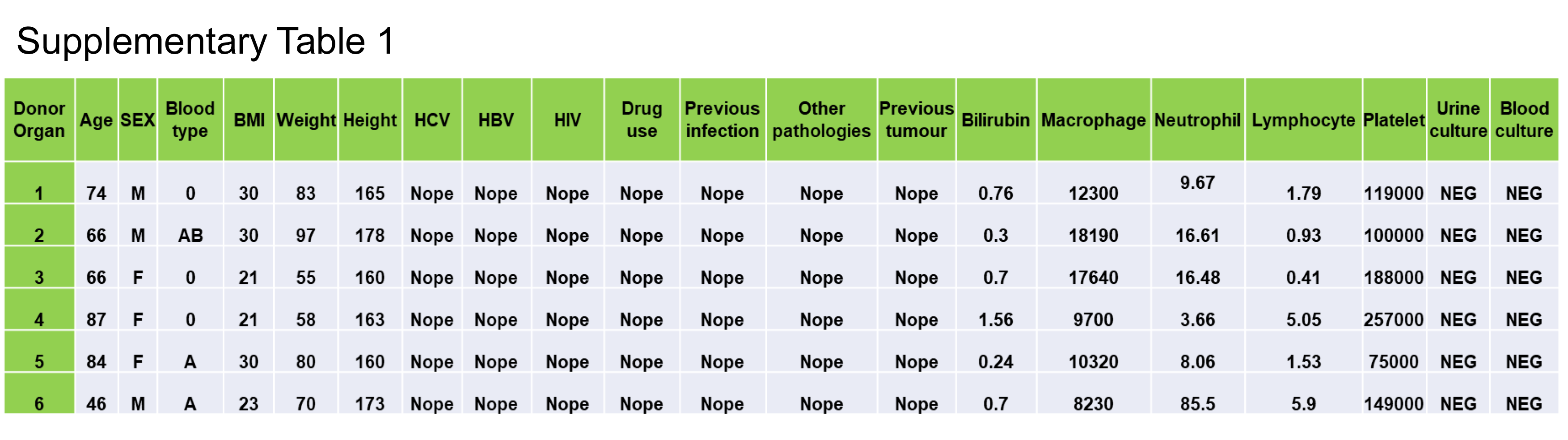

Supplement: Supplementary file 1 [file cells-08-01443-s001.zip › cells-595767-supplementary.tif]
